# Assessment of proprietary and improvised pelvic binders: time to application and displacement during casualty evacuation

**DOI:** 10.1136/military-2024-002865

**Published:** 2025-04-15

**Authors:** Thomas John Howe, H Claireaux, G Morgan, L McMenemy, S D Masouros, A Ramasamy

**Affiliations:** 1 Imperial College London Department of Bioengineering, London, UK; 2 Academic Department of Military Trauma and Orthopaedics, Royal Centre for Defence Medicine, Birmingham, UK

**Keywords:** TRAUMA MANAGEMENT, Trauma management, ACCIDENT & EMERGENCY MEDICINE, Orthopaedic & trauma surgery

## Abstract

**Introduction:**

Pelvic injuries resulting from high-energy trauma have an approximately 10% mortality rate. Suspected pelvic injuries are treated with pelvic binders to stabilise fractured bones and promote tamponade until surgical treatment. The effectiveness of pelvic binders in reducing mortality risk depends on accurate positioning of the binder. This study quantifies the ability of proprietary and improvised pelvic binders to maintain their positioning during a simulated casualty evacuation.

**Methods:**

One improvised and three proprietary pelvic binders were tested in their ability to maintain their initial placement. Combat Medical Technicians applied binders to healthy subjects and then performed a simulated casualty evacuation. The time taken to apply each binder was measured. The evacuation consisted of: 20 m casualty drag, transfer onto a soft stretcher, 100 m evacuation on a soft stretcher, transfer onto a rigid stretcher and transfer into a field ambulance. Binder placement was measured using bony landmarks after the initial positioning and after each phase of the simulated casualty evacuation.

**Results:**

The field-expedient pelvic splint (FEPS), SAM Pelvic Sling II (SAM) and T-POD Pelvic Stabilisation Device (TPOD) all remained within 45 mm of vertical displacement from their initial placement, which is considered an acceptable range for optimal binder functionality. The Prometheus Pelvic Splint (PROM) fell within this range in 83% of trials. The SAM was the fastest binder to apply, followed by the TPOD, and then the FEPS and PROM which took similar times to apply.

**Conclusions:**

Binders were mostly able to maintain their positioning during the simulated casualty evacuations carried out in this study but differed in their application times. The improvised binder (FEPS) performed comparably to the proprietary binders tested, and its low cost and weight make it a good alternative to proprietary binders in the austere environment.

**WHAT IS ALREADY KNOWN ON THIS TOPIC**

- Pelvic binding devices are effective in the early stabilisation of unstable pelvic fractures.
- Maintaining correct binder positioning around the greater trochanters is essential for successful stabilisation.

**WHAT THIS STUDY ADDS**

- This study quantifies the ability of different pelvic binders to maintain correct positioning during simulated casualty evacuations.

**HOW THIS STUDY MIGHT AFFECT RESEARCH, PRACTICE OR POLICY**

- The field expedient pelvic splint can maintain correct positioning during simulated casualty evacuations and is an effective alternative to proprietary binders in the prehospital environment.

## Background

 Pelvic injuries have notable morbidity and mortality rates of approximately 10%.^1^ The causes of these injuries in civilian populations vary. Low-energy trauma is often frequently observed in elderly patients. This contrasts with high-energy trauma in younger individuals, such as road-traffic collisions or falls from height. In military contexts, pelvic injuries predominantly result from high-energy traumas due to insults such as blast. Notably, the mortality rates associated with pelvic fractures in military populations surpass those in civilian contexts, reaching 60.8% in recent conflicts.^2^ The primary contributor to mortality in these cases is non-compressible haemorrhage.^2 3^

Mortality associated with pelvic injury is attributable to bleeding from arterial, venous and osseous injuries.^4^ Notably, the loss of bony containment allows the pelvic volume to expand, heightening the risk of significant haemorrhage and reducing the likelihood of effective tamponade.^5^ This can lead to hypovolaemic shock and potentially fatal outcomes.

The prehospital treatment of pelvic injury aims to reduce pelvic volume to facilitate blood-clot formation and control bleeding.^6^ Pelvic binding devices aim to compress the femoral greater trochanters medially and maintain pelvic stability for evacuation to higher levels of care.^7^ The use of pelvic binders to treat unstable pelvic fractures is recommended by both Advanced Trauma Life Support (ATLS) and UK National Institute for Health and Care Excellence (NICE) guidelines.^8 9^ Biomechanical cadaveric^10–13^ and clinical^14 15^ studies have demonstrated the effectiveness of pelvic binders.

Proper positioning of pelvic binders is essential for successful stabilisation of pelvic fractures. Binders must apply force to the femoral greater trochanters to effectively close the pelvic diastasis gap. A retrospective study on the effect of pelvic binder placement, measured using medical imaging, found that improperly placed pelvic binders led to an average residual diastasis gap 2.8 times larger compared with correctly positioned binders.^6^ It is unknown whether this deviation from ideal placement occurred at application or during patient evacuation, as measurements were taken from CT scans taken when the patients arrived at a field hospital. Based on the retrospective study methodology, anthropomorphic data and binder geometry: proper ‘trochanteric’ placement corresponds approximately to a vertical binder movement of less than 45 mm in either direction from an original position over the trochanteric region of the femur.^6 16^

Our previous laboratory testing of pelvic binders measured the ability of improvised and proprietary binders to hold a force sufficient to reduce an open book pelvic fracture.^17^ In addition to being able to maintain a sufficient force, pelvic binders must also be quick to apply and be able to remain within the effective trochanteric region, especially in the prehospital environment and during evacuation in the field. This allows any haematoma that has formed to remain stable and reduces the risk of further bleeding.

This study aimed to analyse proprietary and improvised pelvic binders in their application time and ability to maintain trochanteric placement in the field during simulated casualty evacuations. An enhanced understanding of existing treatment options can inform policy, clinical decision-making, and guide future developments in pelvic injury management.

## Methods

### Materials

**Figure 1:**
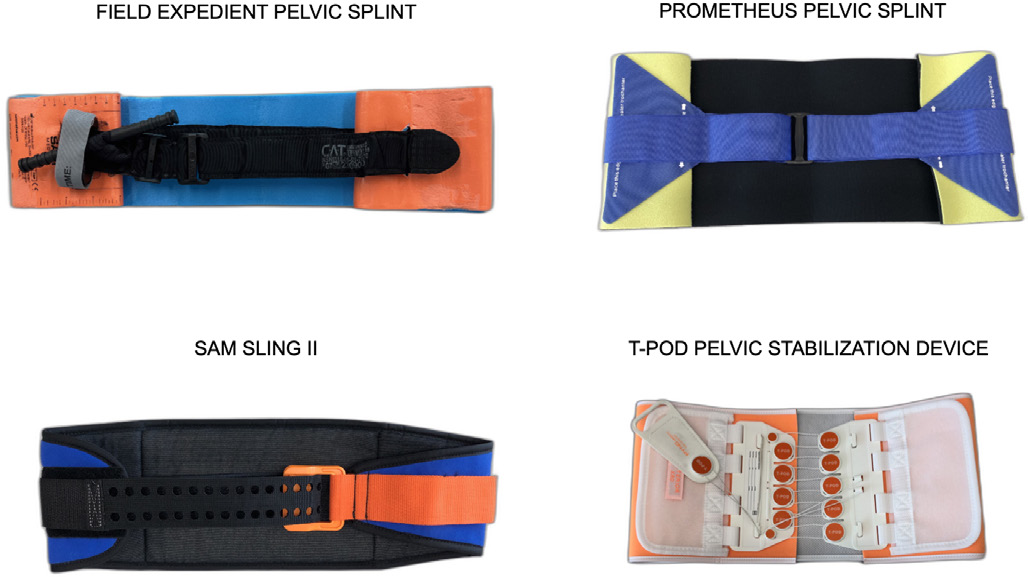
Photograph demonstrating the pelvic binders evaluated.

All phases of the study took place during routine military-medical training. Three proprietary binders were evaluated: the SAM Pelvic Sling II (SAM; SAM Medical, Tualatin, Oregon, USA), the Prometheus Pelvic Splint (PROM; Prometheus Medical, Herefordshire, UK) and the T-POD Pelvic Stabilisation Device (TPOD; Teleflex, Wayne, Pennsylvania, USA). Additionally, the improvised ‘field-expedient pelvic splint’ (FEPS)^18^ was evaluated as it demonstrated comparable mechanical abilities to proprietary binders during laboratory testing.^17^ The FEPS was constructed from the Combat Application Tourniquet (C-A-T Resources, LLC, Rock Hill, South Carolina, USA) and the SAM splint (SAM Medical, Tualatin, Oregon, USA) (figure 1)

### Device evaluation

To quality assure the method two military doctors (TJH and HC) ensured that six British Army Combat Medical Technicians (CMTs) could correctly apply each pelvic binder (SAM, PROM, TPOD and FEPS). Each of the CMTs then performed a treatment and simulated evacuation with each of the four pelvic binders, totalling 24 trials with 6 trials per pelvic binder. This occurred as part of a routine exercise with six unique clinical scenarios requiring utilisation of the Battlefield ATLS (BATLS) paradigm. Each clinical scenario was used once per binder and rotated among the CMTs to prevent repeats.

**Figure 2:**
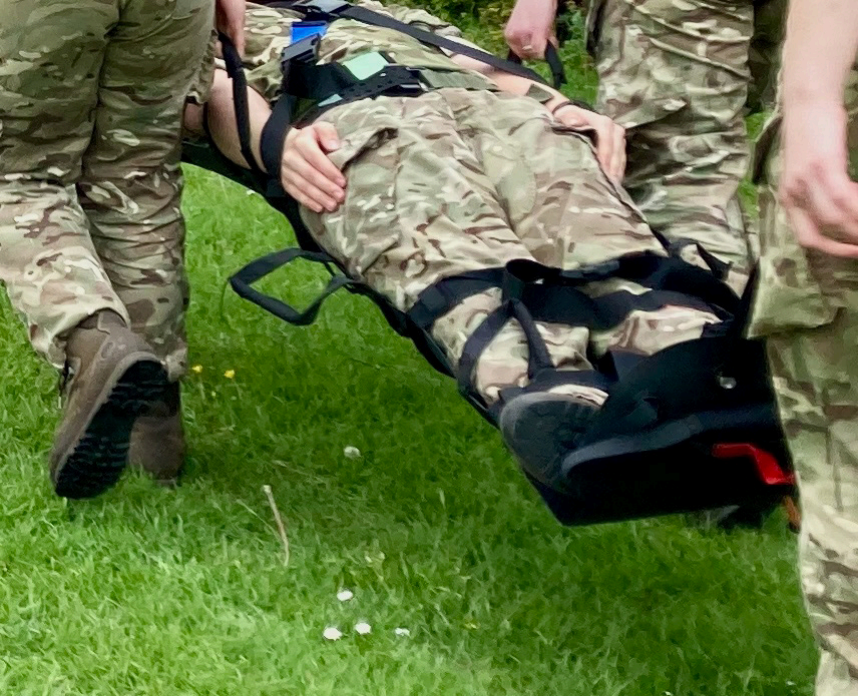
Photograph demonstrating simulated casualty extraction on soft stretcher.

Time to application was recorded from the opening of the pelvic binder packaging to correct application. For the FEPS, this was timed from the point at which the CMT commenced improvising the binder to correct application. Each trial consisted of a 20 m casualty drag, 100 m evacuation on a soft stretcher (figure 2) and transfer onto a rigid stretcher into a vehicle. The correct binder position was confirmed by TJH or HC prior to commencing the simulated casualty evacuation.

**Figure 3:**
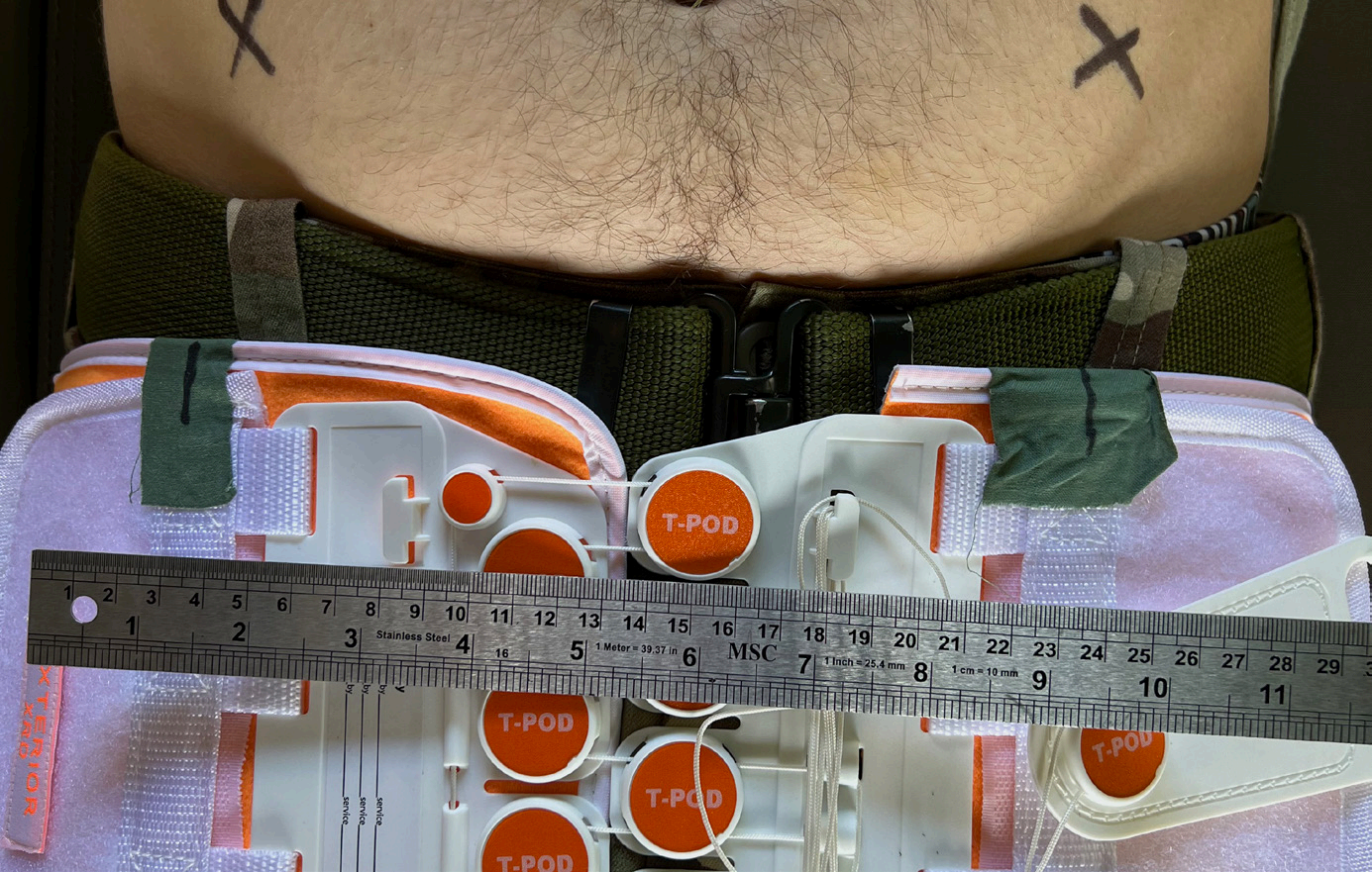
Photograph demonstrating the images taken for binder evaluation: The anterior superior iliac spines are marked with an X, the binder marked with a vertical line and a 30 cm ruler included for scale.

Photographs were used to assess the maintenance of pelvic binder position (figure 3). The anterior superior iliac spines (ASIS) were palpated by TJH and confirmed by HC. The position was marked with an X using a marker pen. The binders were marked with a vertical line using a marker pen. A 30 cm metal ruler was placed on the abdomen for each photograph to allow images to be calibrated for analysis, and to ascertain any error in measurement. The camera was held orthogonally to the patient’s abdomen 1 m away. Photographs were taken immediately after binder application and after each phase of the evacuation.

### Image analysis

Images were analysed in ImageJ (v.1.54, ImageJ, US National Institutes of Health, Bethesda, Maryland, USA) using the left and right ASIS as bony landmarks, and a ruler for scale. Two markers were applied to each binder and their vertical and horizontal displacements relative to the bony landmarks were calculated, where the horizontal direction was defined as the direction of the line connecting the two bony landmarks (figure 3).

### Statistical analysis

Application times were compared using a one-way analysis of variance (ANOVA) test with Tukey’s honestly significant difference (HSD) test. Maximal displacement was recorded for each trial. The displacement data for each binder were tested using a one-sample, one-tailed Student’s t-test against a null hypothesis of 45 mm, with a Bonferroni correction factor of 4. Horizontal displacement was compared using a one-way ANOVA.

A null hypothesis of 45 mm was chosen based on the width of the ‘trochanteric’ region (w*_t_*) and the distance between the central buckle springs of the Sam Sling II (w_s_).^16^ The vertical distance from a binder placed perfectly in the centre of the ‘trochanteric’ region to the threshold described by Bonner *et al* can be calculated as 0.5 w*_t_*+0.5 w_s_, which is approximately 45 mm.^6^

## Results

### Application times

**Figure 4:**
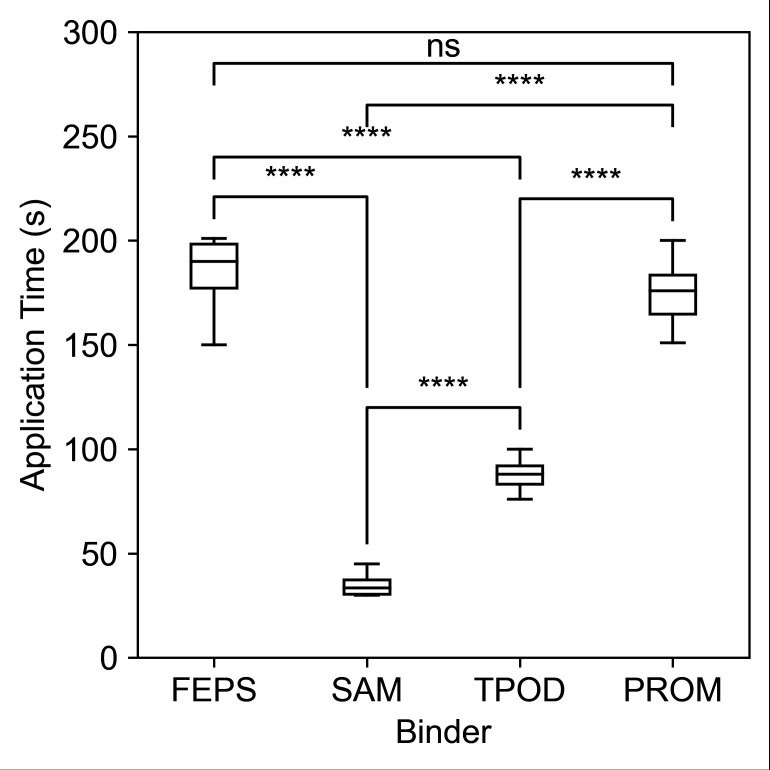
Box and whisker plots of application times for each pelvic binder tested, and results of a one-way ANOVA test with Tukey’s HSD. n=6. ****denotes p<0.0001; ns denotes no statistical significance. ANOVA, analysis of variance; FEPS, field-expedient pelvic splint; HSD, honestly significant difference; PROM, Prometheus Pelvic Splint; SAM, SAM Pelvic Sling II; TPOD, T-POD Pelvic Stabilisation Device.

The application times for the trials of each binder and the results of a one-way ANOVA test with Tukey’s HSD are shown in figure 4. The means and SD of application time for each binder (in seconds) were 184±19, 35±6, 88±8 and 175±17 for the FEPS, SAM, TPOD and PROM, respectively. The SAM took less time to apply than each of the other binders (p<0.0001). The TPOD took less time to apply than each of the FEPS and PROM (p<0.0001). The FEPS and the PROM had no significant difference in their application times.

### Binder displacement

**Figure 5:**
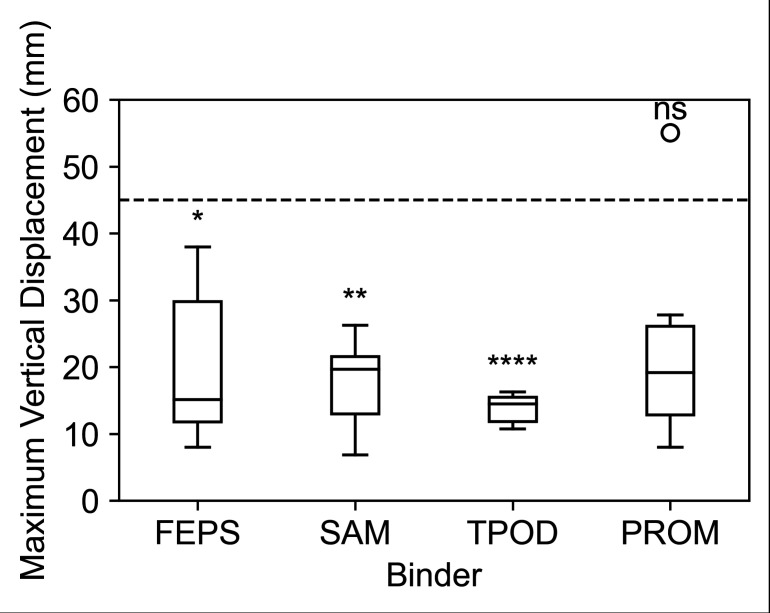
Box and whisker plots of the maximum vertical displacements of each trial for each of the four tested binders, and results of a one-sample, one-tailed Student’s t-test against a null hypothesis of a threshold of 45 mm vertical displacement. n=6. *0.01<p<0.05; **0.001<p<0.01; ****p<0.0001; ns=no significance. P values include a Bonferroni correction factor of 4. FEPS, field-expedient pelvic splint; PROM, Prometheus Pelvic Splint; SAM, SAM Pelvic Sling II; TPOD, T-POD Pelvic Stabilisation Device.

The maximum vertical displacements for the trials of each binder are shown in figure 5 along with the significance of each binder’s t-test against a null hypothesis displacement of 45 mm. Vertical and horizontal displacement for each binder is shown in table 1. The FEPS, SAM and TPOD were all found to have maximum vertical displacements significantly less than 45 mm (Bonferroni adjusted: p<0.01, p<0.001, p<0.0001, respectively). The PROM maximum vertical displacement was not significantly less than 45 mm (Bonferroni adjusted p>0.05). There was no significant difference in horizontal displacement between binders.

**Table 1:** Mean maximal displacements. Vertical displacement compared with a one-sample, one-tailed Student’s t-test against a null hypothesis of 45 mm, with a Bonferroni correction factor of 4. Horizontal displacement compared using a one-way ANOVA test.

Binder	Vertical			Horizontal		
	Mean maximal displacement (mm)	SD of maximal displacement (mm)	P value (incl. Bonferroni)	Mean maximal displacement (mm)	SD of maximal displacement (mm)	P value
FEPS	25.00	11.76	<0.01	12.10	5.99	>0.05
SAM	22.67	9.99	<0.001	10.54	10.67	
TPOD	17.50	5.08	<0.0001	9.56	5.20	
PROM	28.00	15.80	>0.05	8.58	5.70	

FEPS, Field Expedient Pelvic Splint; incl., including; PROM, Prometheus Pelvic Splint; SAM, SAM Pelvic Sling II; TPOD, T-POD Pelvic Stabilisation Device.

## Discussion

The FEPS, SAM and TPOD all had maximum vertical displacements of less than 45 mm (p<0.01, p<0.001, p<0.0001, respectively), indicating that these binders are capable of maintaining proper trochanteric position during simulated casualty evacuations. The TPOD was the least variable in terms of maximum vertical displacements in its trials. The PROM maximum vertical displacement was not significantly less than 45 mm (Bonferroni adjusted p>0.05); however, only one trial of the six fell above this threshold.

The FEPS was able to maintain a maximum vertical displacement of less than 45 mm (p<0.01) despite being an improvised device, the FEPS had an application time which was not significantly different from the PROM, a proprietary device (p>0.05). This is notable because this includes the time for the FEPS to be produced from other items of military medical equipment. These results indicate that the FEPS is a viable treatment option if there are not enough proprietary pelvic binders readily available to treat all suspected pelvic fracture patients, as may be the case in an austere environment or a resource-limited mass-casualty scenario.

The SAM was faster to apply than all other pelvic binders (p<0.0001). This is likely attributable to the simplicity of its application method. Prior practice in applying the SAM in the medical training of the six CMTs performing the treatments may have also been a contributing factor. However, the PROM is currently in routine use in the British Defence Medical Services, so the effect of prior training was not unique to the SAM.

There is published cadaveric work comparing proprietary and improvised pelvic binders.^19 20^ There are no studies involving simulated patient evacuation, or the FEPS. This study complements biomechanical analyses of these devices.^17 21^ Significant vertical displacement of a pelvic binder from the trochanteric region is likely to affect the circumferential tension at the pelvic ring required to control bleeding. Poor positioning away from the trochanters has been shown in a radiological study to affect reduction of the injured pelvis.^6^

A limitation of the findings of this study is the small number of trials performed per pelvic binder. A higher-powered study would likely improve the strength of any findings. The field-fidelity of the simulated casualty evacuation on patients with an uninjured pelvis may represent a limitation. Adding realistic transport to a point of care such as a field hospital could enhance the findings of this study. Similarly, conducting the trials with the additional challenge of inclement weather, poor visibility and physical/cognitive fatigue would improve the transferability of these findings to the military operational environment.

The accuracy of the image-capture and data-analysis methods used are well-suited for two-dimensional applications but are susceptible to errors in three-dimensional space. The error of these methods was calculated as being less than ±6 mm. This was calculated by calibrating the system on the distance between the 0 cm and 20 cm marks on the ruler, and the 5 cm and 25 cm marks for one trial, chosen at random, on each binder. The maximum difference between these two measurements was calculated for each binder.

We chose not to control binder application tension, as it was felt this replicates real-world practice and the inherent interoperator variability when following recommended instructions for use. A future trial could use instrumented pelvic binders to track tensile force and positioning throughout the trial. This would allow for increased accuracy and frequency of data capture, and enable data capture at the time of application and mid-movement; for example, binder position could be reported during the 20 m casualty drag, rather than before and after. This may help quantify clinical risk/benefits of specific binders and aid further policy decision-making.

## Conclusions

Four pelvic binders were assessed in their application times and ability to maintain trochanteric position during a simulated casualty evacuation. Of the 24 total trials, only one trial resulted in a maximum vertical displacement of greater than 45 mm, thus considered falling outside of the trochanteric region. These findings suggest that poor binder positioning is more likely to result from incorrect initial placement than movement during patient evacuation.

The improvised pelvic binder, FEPS, was able to hold trochanteric position, had a similar application time as a proprietary binder, and prior work has shown that it also holds the intended initial closure force; hence, it presents a viable treatment solution in austere environments where a proprietary binder may not be available or may have been used already on another patient.

The main limitation of this study is the small number of trials performed per device and that it stops short of simulating transport of the patient to a treatment facility. A future study should be performed with a greater number of trials, in a complete evacuation procedure, and with instrumented pelvic binders to increase the accuracy of data capture.

## Data Availability

Data are available upon reasonable request.
